# Diversity of Disorders Causing Neonatal Cholestasis – The Experience of a Tertiary Pediatric Center in Germany

**DOI:** 10.3389/fped.2014.00065

**Published:** 2014-06-23

**Authors:** André Hoerning, Simon Raub, Alexander Dechêne, Michelle N. Brosch, Simone Kathemann, Peter F. Hoyer, Patrick Gerner

**Affiliations:** ^1^Department for Pediatric Nephrology, Gastroenterology, Endocrinology and Transplant Medicine, Clinic for Pediatrics II, University Children’s Hospital Essen, University Duisburg-Essen, Essen, Germany; ^2^Department of Pediatrics and Adolescent Medicine, University Children’s Hospital Erlangen, Friedrich-Alexander University, Erlangen, Germany; ^3^Department of Gastroenterology and Hepatology, University Hospital Essen, University Duisburg-Essen, Essen, Germany; ^4^Department of Pediatrics and Adolescent Medicine, University Children’s Hospital, Freiburg, Germany

**Keywords:** neonatal cholestasis, neonatal jaundice, biliary atresia, Kasai procedure, ERCP

## Abstract

**Background and Objective:** Rapidly establishing the cause of neonatal cholestasis is an urgent matter. The aim of this study was to report on the prevalence and mortality of the diverse disorders causing neonatal cholestasis in an academic center in Germany.

**Methods:** Clinical chemistry and cause of disease were retrospectively analyzed in 82 infants (male *n* = 42, 51%) that had presented with neonatal cholestasis to a tertiary medical center from January 2009 to April 2013.

**Results:** Altogether, 19 disorders causing neonatal cholestasis were identified. Biliary atresia was the most common diagnosis (41%), followed by idiopathic cases (13%), progressive familial intrahepatic cholestasis (PFIC, 10%), cholestasis in preterm infants (10%), α1AT deficiency, Alagille syndrome, portocaval shunts, mitochondriopathy, biliary sludge (all 2%), and others. Infants with biliary atresia were diagnosed with a mean age of 62 days, they underwent Kasai portoenterostomy ~66 days after birth. The majority of these children (~70%) received surgery within 10 weeks of age and 27% before 60 days. The 2-year survival with their native liver after Kasai procedure was 12%. The time span between Kasai surgery and liver transplantation was 176 ± 73 days. Six children (7%), of whom three patients had a syndromic and one a non-syndromic biliary atresia, died prior to liver transplantation. The pre- and post-transplant mortality rate for children with biliary atresia was ~12 and ~17%, respectively.

**Conclusion:** Neonatal cholestasis is a severe threat associated with a high risk of complications in infancy and it therefore requires urgent investigation in order to initiate life saving therapy. Although in the last 20 years new causes such as the PFICs have been identified and newer diagnostic tools have been introduced into the clinical routine biliary atresia still represents the major cause.

## Introduction

Neonatal jaundice lasting longer than 14 days after birth is unusual. In some of these infants it is associated with breast feeding (1), but cholestasis must always be excluded by measurement of the conjugated bilirubin in addition to the total bilirubin. Neonatal cholestasis is a serious condition requiring immediate further investigation because it may be due to life-threatening disorders that must be addressed early in order to rapidly initiate specific treatment (2).

The incidence of neonatal cholestasis is ~1 in 2500 live births (3, 4). It results from impairment in bile excretion, caused by various disorders such as defects of intrahepatic production or transmembrane transport of bile, or a mechanical ductal obstruction preventing bile flow. Of the various conditions that can present with neonatal cholestasis, biliary atresia is the most common cause throughout the world and has been reported to account for one-third of the cases (5–7). Early diagnosis is particularly important in biliary atresia because timely surgical intervention with Kasai portoenterostomy correlates with better long-term outcome and patient survival (8–10). However, the diagnosis of cholestatic jaundice in neonates is often delayed and this may result in a poor patient outcome.

Here, we report the retrospective analysis of 82 infants with neonatal cholestasis who were presented to a single tertiary center during a 4-year period. This report provides information on the prevalence and mortality of the various causes of neonatal cholestasis, the diagnostic algorithm, and the timing of specific medical and surgical intervention in infants with biliary atresia in this cohort.

## Patients and Methods

### Patient cohort

A cohort of infants referred with the diagnosis of neonatal cholestasis during a 4-year period from January 2009 to April 2013 to Essen, a tertiary medical center in western Germany, was retrospectively analyzed. Fifteen children referred from Eastern Europe (Russia *n* = 1, Czech Republic *n* = 3, Hungary *n* = 11) during this period were excluded for demographical reasons. These infants were already diagnosed for neonatal cholestasis, those with biliary atresia received a hepatoportojejunostomy in their home countries and were transferred solely for the liver transplantation.

Neonatal cholestasis was defined as neonatal prolonged jaundice with conjugated hyperbilirubinemia exhibiting a conjugated (direct) bilirubin of more than 1 mg/dL (in combination with a total bilirubin of <5.0 mg/dL or a direct bilirubin fraction of >20% of the total). After 4 weeks, a direct bilirubin >0.2 mg/dL was used as a cut-off value. All cholestatic infants received immediate supplementation with lipid-soluble vitamins and parenteral vitamin K supplement in case of coagulopathy.

### Standard diagnostic work-up for conjugated hyperbilirubinemia

Infants transferred with neonatal cholestasis underwent a standard diagnostic protocol, which included a detailed history and physical examination, hematological, biochemical and serological investigations, imaging procedures, and liver biopsy. All medical interventions and laboratory diagnostics as well as the clinical procedures were performed in accordance with the declaration of Helsinki.

Clinical standard procedures such as a precise physical examination (e.g., facial dysmorphic stigmata in Alagille syndrome), pulse oximetry, abdominal ultrasonography, echocardiography, ECG, and thoracic X-ray (butterfly vertebrae) were performed in all children. Full blood cell count (including reticulocytes), a blood microscopy, clinical chemistry including direct and indirect bilirubin, serum aminotransferases (ALT, AST, and GGT), albumin, electrolytes, creatinine, urea, uric acid, iron level, ferritin, transferrin saturation, creatinkinase, lipase, alpha fetoprotein, triglycerides, cholesterol, immunoglobulins (IgM, IgG, and IgA), serum bile acids, and a venous blood gas analysis were performed in each patient. In cases of anemia, serum levels of LDH and haptoglobin were determined and a direct Coombs test was performed in order to differentiate in between immune and non-immune mediated hemolysis. Coagulation parameters including INR, aPTT, fibrinogen, and antithrombin III were assessed in each patient. To exclude an infectious cause for cholestasis, a virological analysis of all hepatotropic viruses (e.g., CMV, EBV, HSV, HAV, HBV, HCV, Parvovirus B19, and others) as well as bacterial and parasitic causes were considered. Endoscopic retrograde cholangiopancreatography (ERCP) was performed in infants with neonatal cholestasis, acholic stool for more than 7 days and a small or even absent gallbladder in ultrasonography.

Since cystic fibrosis and alpha-1-antitrypsin (α1AT) deficiency can mimic extrahepatic biliary atresia, both disorders were also excluded before ERCP procedure. Cystic fibrosis was diagnosed by determination of fecal elastase or immunoreactive trypsin (infants younger 4 weeks of age). α1AT levels were determined in serum and if reduced the deficient variant of the α1AT protein was identified by protease inhibitor (PI) typing using polyacrylamide isoelectric focusing (PI-M, PI-S, PI-Z alleles) (11).

In cases, biliary atresia was unlikely or excluded the following tests were performed: to rule out an endocrinological cause, morning cortisol level, IGFBP3, IGF1, and TSH plus free thyroxine and triiodothyronine were assessed. Metabolic diseases were diagnosed by determining urinary and serum amino acid profiles (to detect, e.g., tyrosinemia type I), organic acids in urine (e.g., methylmalonic or propionic acidemia), acylcarnitine profile in blood (fatty acid oxidation disorders), VLCFA levels (peroxisomal disorders such as Zellweger’s syndrome), serum glucose and lactate (pre- and postprandial to detect mitochondrial disorders), and ammonium in plasma (urea cycle disorders such as ornithine transcarbamylase deficiency). The investigation for carbohydrate metabolism disorders (e.g., galactosemia) is included in the newborn screening panel. These metabolic studies were all performed at the Laboratory for Metabolic Diseases, University Hospital Heidelberg, Germany. To achieve the diagnosis of inherited disorders of bile acid synthesis, urinary cholanoids (bile acids and bile alcohols) were analyzed as reviewed by Clayton et al. (12). A bone marrow aspiration was performed in infants with splenomegaly if a metabolic disease (e.g., Gaucher or Niemann–Pick disease), leukemia/lymphoma or histiocytosis was suspected. Since autoimmune mediated cholestasis is a very rare cause, levels of ANA, anti-dsDNA, and anti-Ro/SSA antibodies were determined in neonates either from mothers with a previous history of systemic lupus erythematodes or as a last diagnostic option before terming it idiopathic.

Liver biopsy was carried out in all patients except in those infants that had unequivocal findings on other tests, especially ERCP. The liver biopsy was examined with standard histology and electron microscopy (storage diseases), immune histology (to diagnose PFIC I–III, Professor R. Kubitz, Dusseldorf, Germany), and – if clinically suspected – also for respiratory chain disorders (shock frozen biopsy, MGZ, Munich, Germany). For histological diagnosis of biliary atresia, standard criteria (e.g., bile ductular proliferation, portal tract fibrosis, and bile plugs in portal triads) were used (13). Neonatal giant-cell hepatitis (NGCH) was defined as intrahepatic cholestasis with presence of characteristic giant-cell lesions and idiopathic cases when no other detectable cause was identified.

### ERCP procedure

A pediatric videoendoscope with an outer diameter of 7.5 mm was used for ERCP (Olympus PJF160 7.5, Olympus Co., Tokyo, Japan). Informed consent was obtained from all parents. All examinations were performed under general anesthesia with continuous monitoring of vital functions. The children received intravenous antimicrobial therapy for at least 24 h after cholangiography to prevent persistent bacteremia and cholangitis. Blood cell count and clinical chemistry were assessed 4–6 h after ERCP, whilst serum amylase and lipase were routinely measured the following day.

### Statistical analyses

The data analysis includes descriptive statistics of means with standard deviations, medians, and ranges. Patient groups were compared using the Mann–Whitney *U* test. All analyses were performed using GraphPad Prism V5.0a. A *p* value <0.05 was considered statistically significant.

## Results

### Patient demographics

A total population of 82 infants (male *n* = 42, 51%) was assessed within the period of analysis. In the majority of children (*n* = 49, 60%) the diagnosis was already secured: these children were referred for further treatment and/or liver transplantation. Thirty-three infants (40%) with cholestasis of unknown origin were transferred from local community hospitals for further diagnostic work up in our institution.

**Figure 1:**
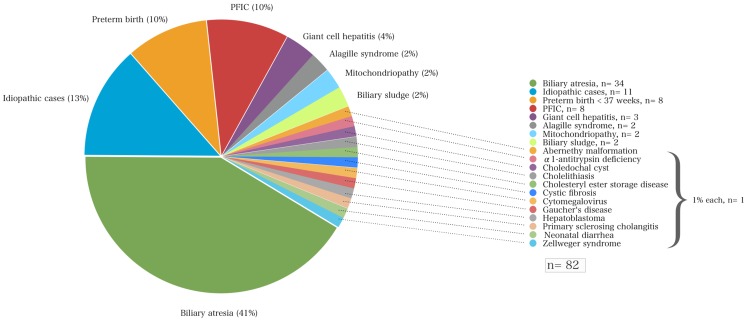
**The various disorders that led to neonatal cholestasis and the proportion of patients of each entity (%) of a cohort of 82 infants at a single tertiary medical center**.

Of the total cohort, the mean age at initial referral to our center was 71 ± 81 days (range 5–558 days). Most (87%) of these 82 children were referred from hospitals within 250 km of Essen, other than one child from Munich (640 km distance). The most common underlying liver disease was biliary atresia (*n* = 34; 41%) followed by idiopathic cases (*n* = 11; 13%), progressive familial intrahepatic cholestasis (PFIC, *n* = 8; 10%), and cholestasis secondary to total parenteral nutrition in preterm infants (*n* = 8; 10%). Among the 34 children with biliary atresia 4 (12%) had a syndromic variant defined by polysplenia, a situs inversus, or unusual vascular anomalies such as absence of an inferior vena cava and a preduodenal portal vein. Among the eight infants diagnosed for PFIC, seven suffered from subtype II and one infant from type III. The onset of cholestasis in preterm infants was in the majority of the cases early after birth, in three cases it occurred time-delayed (67 ± 23 days). None of these infants had neonatal (bacterial or viral) infections. Neonatal cholestasis in preterm infants was regressive in all cases. Laboratory chemistry normalized 160 ± 87 days after birth (median 149, range 85–338 days). More detailed information on other disease entities is shown in Figure 1.

**Figure 2:**
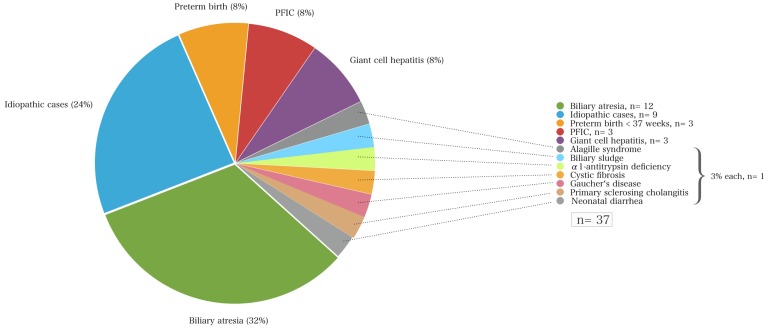
**The causes of neonatal cholestasis and the proportion of patients of each entity (%) of a cohort of 37 infants that were initially referred with unclear cholestasis to the University Children’s Hospital Essen**.

Infants with neonatal cholestasis of unclear origin that had been referred for diagnostic work-up to our center (*n* = 37) had biliary atresia (*n* = 12; 32%) followed by idiopathic cases (*n* = 9; 24%), PFIC (*n* = 3; 8%), cholestasis secondary to total parenteral nutrition in preterm infants (*n* = 3; 8%), giant-cell hepatitis (*n* = 8, 27%), Alagille syndrome, biliary sludge, α1AT deficiency, cystic fibrosis, Gaucher’s disease, and congenital neonatal diarrhea (each *n* = 1, 3%, Figure 2).

### Biliary atresia: Age at hepatoportoenterostomy and at liver transplantation

The mean age at initial referral of infants with biliary atresia was 37 ± 30 days (median 32, range 2–110 days) and when referred to our center the children were 107 ± 112 days (median 82, range 30–558 days) old. Again, most of the children (65%) were referred from hospitals within an area of 250 km around Essen: Essen (*n* = 4), Cologne (*n* = 6), Krefeld (*n* = 4), Duisburg (*n* = 2), Moers (*n* = 3), Dortmund (*n* = 3), and others. The most distant center was Munich (*n* = 1).

Infants were diagnosed for biliary atresia with a mean age of 62 ± 16 (median 60 days, range 32–81 days); they received a hepatoportoenterostomy with a mean age of 66 ± 24 days (median 60 days, range 37–146 days). The 2-year survival with their native liver after Kasai procedure was 12% (4/34 children). The time span between Kasai surgery and liver transplantation was 173 ± 75 days (median 155, range 80–359 days). The mean age at liver transplantation in children with biliary atresia was significantly earlier (229 ± 77 days, median 222, range 87–441 days) compared to all other cases of neonatal cholestasis (525 ± 240 days, median 538, range 241–783 days; *p* < 0.01).

### Outcomes of endoscopic retrograde cholangiopancreatography

In our hospital, ERCP was performed in 22 patients with suspected biliary atresia (see Table 1). In 10 children biliary atresia was excluded by cholangiography (extrahepatic biliary ducts without pathologic findings in 9, sclerosing cholangitis in 1). In 12 children, biliary atresia was either confirmed by typical morphologic changes seen by cholangiography (*n* = 6) or suspected due to absent opacification of extrahepatic biliary ducts after injection of contrast medium (*n* = 6). In the six cases with inconclusive ERCP, additional diagnostic procedures were performed: One patient was diagnosed by hepatobiliary scintigraphy and biopsy and three patients by an intraoperative cholangiography. In the remaining two, unequivocal liver histology plus the detection of a dysplastic gall bladder and increased subcapsular hepatic arterial perfusion on ultrasonography led to the diagnosis of biliary atresia.

**Table 1:** **Results of the ERCP procedure and final diagnoses**.

Patient No.	Diagnosis after ERCP	Final diagnosis
1	Normal biliary tract	Idiopathic
2	Inconclusive	Biliary atresia
3	Normal biliary tract	α1ATD
4	Inconclusive	Biliary atresia
5	Normal biliary tract	CF
6	Inconclusive	Biliary atresia
7	Biliary atresia	Biliary atresia
8	Normal biliary tract	Idiopathic
9	Normal biliary tract	Idiopathic
10	Biliary atresia	Biliary atresia
11	Biliary atresia	Biliary atresia
12	Primary sclerosing cholangitis	Primary sclerosing cholangitis
13	Inconclusive	Biliary atresia
14	Inconclusive	Biliary atresia
15	Inconclusive	Biliary atresia
16	Biliary atresia	Biliary atresia
17	Normal biliary tract	Idiopathic
18	Normal biliary tract	Biliary sludge
19	Biliary atresia	Biliary atresia
20	Normal biliary tract	Idiopathic
21	Biliary atresia	Biliary atresia
22	Normal biliary tract	CESD

*Indication for the ERCP was the presence of a pale, discolored stool, and an inconclusive ultrasonography failing to identify the gallbladder. Inconclusive means that an unequivocal cholangiogram was not obtained during the procedure. CESD, cholesteryl ester storage disease*.

The mean age of investigated children suspected for BA was 50 ± 30 days (median 43, range 17–126) days and mean weight was 4.2 ± 1.0 kg. The average procedure time was 25 min. The overall complication rate was low (9%, *n* = 2). Out of 22 children that underwent ERCP, one suffered from relevant intramural duodenal bleeding and received red blood cell transfusion (15 mL/kg). Another child was reintubated and treated with dexamethasone because of respiratory insufficiency caused by post-interventional laryngotracheal swelling. We observed no cases of post-ERCP pancreatitis.

### Mortality pre- and post-liver transplantation

**Figure 3:**
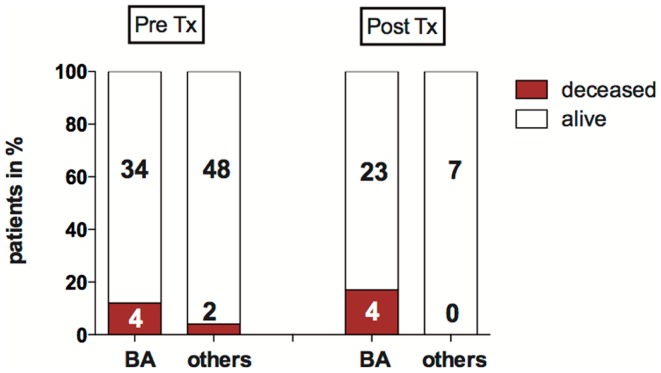
**Mortality pre- and post-LTX in biliary atresia (BA) and other disorders causing neonatal cholestasis**. The bar graph shows the proportion of deceased children before and after liver transplantation (%). Absolute patient numbers are printed in bold inside the bar graph segments.

Children were considered for liver transplantation when they showed liver synthesis insufficiency, hyperbilirubinemia, hypoalbuminemia, dystrophy, clinical sequelae of portal hypertension such as diuretic resistant ascites, and/or recurrent esophageal variceal bleeding. Thirty of the 82 children with neonatal cholestasis required liver transplantation within the period of the study. Biliary atresia was the most frequent indication for transplantation (77%, *n* = 23). Six children among the total study population died before liver transplantation was possible (7%). Of those, three had syndromic variants of BA with malformations such as Abernethy malformation, a congenital heart defect (double outlet right ventricle, DORV), and a Fallots tetrad, one with non-syndromic biliary atresia, one was a preterm infant with a complex malformation syndrome and the other had a Zellweger syndrome. The pretransplant mortality rate for children with biliary atresia was ~12 vs. 4% of children with other disorders causing neonatal cholestasis (Figure 3).

Four children deceased after transplantation: all had biliary atresia, they received Kasai surgery without sufficient biliary drainage 59, 66, 84, and 97 days after birth, respectively. All were suffering from hepatopulmonary syndrome (HPS). In these children, transplantation was performed within 10–19 days after diagnosis of HPS.

In summary, of 82 children with neonatal cholestasis 10 died in the longitudinal course either before or after liver transplantation. The majority of this subgroup with a poor outcome had biliary atresia (*n* = 8), of which four patients had a syndromic BA variant.

## Discussion

A variety of disorders can present with cholestasis during the neonatal period with biliary atresia as the most frequent cause and idiopathic cases as the second common entity. A previous study reported that 35% of infants with neonatal cholestasis had biliary atresia and 30% were considered as idiopathic cases having neonatal hepatitis (5). Other causes included α1AT deficiency (17%), congenital viral infections (9%), Alagille syndrome (6%), and choledochal cysts (3%). Almost 25 years later, this retrospective study confirms these data in part since biliary atresia accounted for about 41% of all infants with neonatal cholestasis that were referred to our hospital (Figure 1; Table 2).

**Table 2:** **The most common disorders causing neonatal cholestasis of the current study compared to those reported in the last 25 years**.

Study	Patients	Country	BA	IC	Metabolic,	PFIC	Preterm	Infection	Alagille	Miscellaneous
			(%)	(%)	endocrine,	(%)	(%)	(%)	(%)	(%)
					genetic (in%)					
Hoerning et al. (14)	82	Germany	41	13	9	10	10	1	2	14
Lee et al. (15)	146	Malaysia	29	38	1	4	5	14	1	8
Aanpreung et al. (16)	252	Thailand	22	23	10	–	18	10	–	17
Bazlul Karim and Kamal (17)	62	Bangladesh	26	24	5	–	–	36	2	7
Stormon et al. (18)	205	Australia	20	25	23	–	20	9	3	0
Yachha et al. (19)	60	India	55	12	3	–	–	8	3	19
Mieli-Vergani et al. (5)	147	England	35	31	17	–	–	9	6	2

*Biliary atresia (BA) and idiopathic cases (IC) are the most common underlying cause of neonatal cholestasis in all studies. Metabolic disorders include genetic and endocrinological causes*.

In contrast to Mieli-Vergani (5), however, the percentage of infants diagnosed with idiopathic cases is lower in the current cohort (13 vs. 30%). This is likely due to the introduction of new diagnostic tools that enable the detection of previously unrecognized conditions. Rates of PFIC and TPN-induced cholestasis have been underestimated in recent years as these disorders were not described in previous demographic studies (Table 2). PFIC was identified in 10% of the cases in this cohort, which is in line with a previous reported empirical evidence of 10–15% of children with neonatal cholestasis (20). The true incidence is not precisely known but is estimated to be 1/50000–1/100000 births (20). Subtypes 1 and 2 represent two-thirds and PFIC3 the remaining third of cases with PFIC (21). Although to date three types of PFIC have been identified (20), only the subtypes PFIC2 (*n* = 7) and PFIC3 (*n* = 1) were diagnosed in this cohort. In addition, the prevalence of α1AT deficiency in the current cohort is low compared to previous reports identifying α1AT deficiency as the third common cause among Caucasians (7).

Recently, molecular genetic tests became available to diagnose the Alagille syndrome, PFIC I-III, and α1AT deficiency (22). These tests have been reported to efficiently identify disease-causing mutations and thus represent an adjunct alternative to timely clear up the cause of cholestasis. Moreover, a more frequent genetic testing for ATP8B1, ABCB11, and ABCB4 would generally identify more cases of PFIC because the immunostaining may namely recognize the relevant proteins but does not detect a loss function mutation – a reason why PFIC may be underdiagnosed in this and current other studies. It thus can be expected that genetic testing will imply the potential to expand the PFIC group among cases of neonatal cholestasis in future studies. In preterm infants, neonatal cholestasis was regressive in all cases. The cause for the development of neonatal cholestasis in these children remains speculative. The majority of preterm neonates developed cholestasis early after birth (63%), a point of time when total parenteral nutrition was just initiated. Alternatively, prematurity in combination with perinatal stress and/or asphyxia as the worst case may have resulted in hepatic dysfunction leading to the perinatal onset of neonatal cholestasis and may represent a potential causal factor for a generally benign and reversible cholestasis in preterm and also at term neonates (23).

In this study, biliary atresia represents by far the most frequent disorder causing neonatal cholestasis (Figure 1; Table 2). However, this study has a limitation. The relative incidences reported here but also in other previous studies (6) need to be handled carefully, since they represent retrospective analyses of single tertiary pediatric transplant centers. The subanalysis of our cohort demonstrates that the study group includes two different groups – one part is the local region’s infants with cholestasis undifferentiated and for further investigation at initial referral, and the second a selected group containing already diagnosed children referred from a wider area for further treatment and/or transplantation (Figures 1 and 2). The latter subset of the group does bias the incidence and mortality of disorders of the whole population to a certain extent because children referred from other centers may represent a grouping of more severe cases whereas some of the cases with more benign cases of neonatal cholestasis would not have been referred. Therefore, the severity of diseases may be skewed toward a more severe spectrum of possible causes of neonatal cholestasis (e.g., biliary atresia) that may generate the differences in the incidences of other population-based studies.

On the other hand is the fact that the Children’s Hospital Essen is located in one of the biggest congested urban area of Germany containing over 5 million habitants. The catchment area is small and the majority of the children (87%) that were referred to our hospital with neonatal cholestasis were from community hospitals from cities within an area of 250 km. Although the consideration of a catchment area may not completely abrogate a possible skewing toward more severe disorders, these children with neonatal cholestasis represent a homogenous population within a well-defined area of West-Germany. The differences with other published series, namely the decrease of idiopathic cases of neonatal cholestasis and the increase of PFIC, may thus be caused at least in part by an increased knowledge of new diagnoses and improvements of the diagnostic work-up.

Neonatal cholestasis is a severe disorder in early childhood with a pretransplant mortality that is not negligible. Especially for children with biliary atresia it was about 12%. Biliary atresia was the most frequent disorder in our and previously reported cohorts (6, 24) leading to liver transplantation. Although only infants with biliary atresia died after transplantation in our cohort the intraoperative complications could have also affected infants with other causes of neonatal cholestasis. The majority of those infants that died before liver transplantation had disorders with an unfavorable prognosis (three with syndromic variants of biliary atresia, one with complex malformation syndrome, and one with Zellweger’s syndrome), only one child had a non-syndromic biliary atresia. However, the fact that syndromic variants seem to carry a higher complication rate may depend on the small series as previous multi-center studies did not find a difference in between syndromic and non-syndromic variants of biliary atresia (25, 26).

It is the late referral of children with biliary atresia that is widely suggested to be accountable for an increased mortality (26–29) since the success rate of the Kasai procedure is closely associated with the age at the time of surgery (9, 10, 30). Besides age at hepatoportoenterostomy several other risk factors for a failure of Kasai surgery have been described and include histologic features (small or absent bile ducts at the portal plate) and recurrent episodes of cholangitis (13). It is reported that children with biliary atresia referred for surgery before 60 days of age do markedly better with a reestablishment of bile flow in more than 80% than those older than 90 days at the time of operation (5, 27, 31–33). This had been shown both in the short-term and in the long-term course (9, 34, 35). The earlier surgery was performed, the later a liver transplantation was usually needed (8). In the short-term, infants that earlier received a Kasai procedure less frequently developed intrapulmonary vascular dilatations, a condition preceding the development of a HPS (14). In the long-term, every second infant with biliary atresia who underwent surgery <30 days of age was still living with his/her own liver 10 years later compared to 15% of those treated after 90 days of age (34). In our cohort, infants with biliary atresia were initially presented to a secondary health care provider with a mean age of 37 days, the mean age at diagnosis was 62 days. This is in line with the average age at diagnosis of biliary atresia, e.g., in the USA (36) and Germany (37) (~60 days). The Kasai procedure was performed soon after diagnosis at a mean age of 66 days demonstrating that the late diagnosis of biliary atresia caused the delay in an appropriate treatment. The majority of the children in this cohort (~70%) underwent surgery before 10 weeks of age and only 27% before 60 days. Hence, corrective surgery within the first 2 months of age is still by far not the routine although substantial efforts have been undertaken to overcome this problem (38, 39).

Among these it is a current endeavor to centralize the services and treat children with biliary atresia in few experienced centers in order to increase surgical success and survival with the native liver (26). A recent large German multi-center study confirmed a relation between outcome and experience of the surgical center and reported a 2-year survival of 20% with their native liver (40). The 2-year survival with the native liver was even less (12%) in our cohort. However, it is important to emphasize that the numbers of this study are biased by the fact that a majority of children with biliary atresia had a history of a failed Kasai procedure and were referred to our center for the purpose of liver transplantation.

The initial and timely detection of biliary atresia is currently and in future will still be a domain of the primary health care provider and depends on the recognition of prolonged jaundice and abnormal stool and/or urine color. It is often challenging as a substantial group of breast fed newborns (ca. 15%) develop benign neonatal jaundice (1) and infants with biliary atresia often appear well during the first 3 weeks after birth. In Germany, at 4–6 weeks of age a visit to a pediatrician is generally recommended and supported by the governmental health insurance system. A routine dipstick test for urinary bilirubin excretion and/or the use of a stool color card at this visit would be an additional non-invasive and simple check to identify cholestatic infants. Furthermore, both types of biliary atresia, the fetal type with early onset and the perinatal type with later onset during the second to fourth weeks of life (6), would be detected at this time point. However, within the last three decades (5) no essential progress had been achieved in early identification of biliary atresia. There is still an urgent need to increase the awareness of prolonged jaundice caused by neonatal cholestasis and of its severity.

When biliary atresia is suspected, ERCP may serve as a reliable, accurate and safe additional beneficial diagnostic tool in unclear cases of children with non-pigmented stool (37, 41, 42). Here, ERCP clarified the diagnosis in 73% of the cases where biliary atresia was suspected (16/22 infants). Both the low complication rate as well as the average procedure time of ERCP was in line with previous studies (37, 43). Hence, ERCP (if conclusive) may represent a safe method expanding the diagnostic repertoire especially 4–6 weeks after birth when histological findings are sometimes not unequivocal (2, 44). Although it is not generally recommended yet we would therefore encourage performing an ERCP and liver biopsy 4–6 weeks after birth at latest if acholic stools are persisting and biliary atresia is suspected. The ERCP procedure however should be performed by operators specifically skilled in pediatric patients in an expert tertiary center in order to guarantee a safe and reliable procedure that then enables to detect biliary tract abnormalities (e.g., biliary atresia or choledochal cysts) with high sensitivity and specificity.

In conclusion, a variety of hepatobiliary disorders with biliary atresia as the major cause can present with cholestasis during the neonatal period. A systemic approach is the key to reliably achieve a rapid diagnosis in order to initiate disease-specific and in many cases life saving therapy. Therefore, any infant presenting with prolonged jaundice at 14 days of age with or without acholic stool must be evaluated by determination of the fractionated bilirubin and if elevated referred to a subspecialist for pediatric hepatology as soon as possible in order to confirm or rule out biliary atresia and other treatable disorders.

## Conflict of Interest Statement

The authors declare that the research was conducted in the absence of any commercial or financial relationships that could be construed as a potential conflict of interest.

## Abbreviations

α1AT, alpha 1-antitrypsin; AFP, alpha-fetoprotein; ALP, alkaline phosphatase; ALT, alanine aminotransferase; ANA, anti-nuclear antibodies; AST, aspartate transaminase; ATIII, antithrombin III; ATT, α1-antitrypsin deficiency; BA, biliary atresia; CF, cystic fibrosis; CMV, cytomegalovirus; EBV, Epstein–Barr virus; ECG, electrocardiogram; ERCP, endoscopic retrograde cholangiopancreatography; GGT, gamma-glutamyltransferase; HAV, hepatitis A virus; HBV, hepatitis B virus; HCV, hepatitis C virus; HCC, hepatocellular carcinoma; HPS, hepatopulmonary syndrome; HSV, herpes simplex virus;IgA, immunoglobuline A; IGF, insulin-like growth factor; IGFBP, insulin-like growth factor binding protein; INR, international normalized ratio; LDH, lactate dehydrogenase; LKM-1, liver/kidney microsomal antibody; MRCP, magnetic resonance cholangiopancreatography imaging; MRI, magnetic resonance imaging; PFIC, progressive familial intrahepatic cholestasis; PTT, partial thromboplastin time; SMA, smooth muscle antibody; TNP, total parenteral nutrition; TSH, thyroid-stimulating hormone; US, ultrasonography; VLCFA, very long chain fatty acids.
